# IGFBP3 impedes aggressive growth of pediatric liver cancer and is epigenetically silenced in vascular invasive and metastatic tumors

**DOI:** 10.1186/1476-4598-11-9

**Published:** 2012-03-08

**Authors:** Ivonne Regel, Melanie Eichenmüller, Saskia Joppien, Johanna Liebl, Beate Häberle, Josef Müller-Höcker, Angelika Vollmar, Dietrich von Schweinitz, Roland Kappler

**Affiliations:** 1 Department of Pediatric Surgery, Dr. von Hauner Children's Hospital, Ludwig-Maximilians-University Munich, 80337 Munich, Federal Republic of Germany; 2 Department of Pharmaceutical Biology, Ludwig-Maximilians-University Munich, 81377 Munich, Federal Republic of Germany; 3 Institute of Pathology, Ludwig-Maximilians-University Munich, 80337 Munich, Federal Republic of Germany

**Keywords:** Hepatoblastoma, Epigenetics, Methylation, Invasion, IGF2

## Abstract

**Background:**

Hepatoblastoma (HB) is an embryonal liver neoplasm of early childhood with a poor prognosis for patients with distant metastases and vascular invasion. We and others have previously shown that the overexpression of *insulin-like growth factor 2 *(*IGF2*), loss of imprinting at the *IGF2*/*H19 *locus, and amplification of *pleomorphic adenoma gene 1 *(*PLAG1*) are common features in HB, suggesting a critical role of the IGF axis in hepatoblastomagenesis. In this study, we investigated the role of the insulin-like growth factor binding protein 3 (IGFBP3), a known competitor of the IGF axis, in pediatric liver cancers.

**Results:**

The *IGFBP3 *gene was highly expressed in normal pediatric livers but was heavily downregulated in four HB cell lines and the majority of HB primary tumors (26/36). Detailed methylation analysis of CpG sites in the *IGFBP3 *promoter region by bisulfite sequencing revealed a high degree of DNA methylation, which is causatively associated with the suppression of *IGFBP3 *in HB cell lines. Consequently, the treatment of HB cell lines with 5-aza-2'-deoxycytidine resulted in DNA demethylation and reactivation of the epigenetically silenced *IGFBP3 *expression. Interestingly, *IGFBP3 *promoter methylation predominantly occurred in metastatic HB with vascular invasion. Restoring *IGFBP3 *expression in HB cells resulted in reduced colony formation, migration, and invasion.

**Conclusion:**

This study provides the first direct evidence that the reactivation of *IGFBP3 *decreases aggressive properties of pediatric liver cancer cells and that *IGFBP3 *promoter methylation might be used as an indicator for vessel-invasive tumor growth in HB patients.

## Background

Hepatoblastoma (HB) represents the most common primary liver tumor in childhood with an incidence of approximately one new case per million children less than 15 years of age [1]. Pathohistologically, HB resembles various stages of the developing liver, showing malignant epithelial cells with fetal and/or embryonal hepatic differentiation and foci of primitive blastemal cells. The mixed HB subtype also contains interspersed mesenchymal elements, such as immature fibrous tissue, spindle cells, and osteoid [1]. Although HB generally responds well to chemotherapy and the prognosis is usually good [2], the outcome of high-risk patients with metastatic tumors or invasion of large hepatic veins is fatal [3,4].

The type 1 insulin-like growth factor receptor and its ligands, IGF1 and IGF2, are upregulated in a variety of human cancers [5]. In pediatric tumors, such as rhabdomyosarcoma, nephroblastoma, and HB, the role of the IGF axis is particularly important [6]. We and others have shown that the fetal growth factor *IGF2 *is upregulated in almost all HB cases [7,8], even though the underlying molecular mechanism is still not understood. This upregulation could be explained in part by the observation that the loss of imprinting at the *IGF2/H19 *locus is evident in approximately 20% of all *IGF2 *overexpressing HB, thus leading to biallelic expression of the gene [9]. Moreover, the amplification and subsequent upregulation of the transcriptional IGF2 activator *PLAG1 *has been described in the majority of HB cases [10]. Collectively, these data suggest that several mechanisms could be responsible for the frequently observed upregulation of *IGF2*, which is characteristic for the molecular pathogenesis of HB.

The insulin-like growth factor binding protein 3 (IGFBP3) is a multifunctional protein predominantly produced by the liver, which mediates the growth suppression and induction of apoptosis by binding insulin-like growth factors [11]. Accordingly, *IGFBP3 *transgenic mice exhibit a significant reduction in both birth weight and litter size, with a reduction in some organ weights [12]. The stable transfection of *IGFBP3 *results in reduced growth rates of non-small cell lung cancer cells, both *in vitro *and *in vivo*, as xenotransplants in nude mice [13]. Moreover, the addition of recombinant IGFBP3 results in the massive induction of apoptosis, as shown in colon and prostate cancer [14,15]. Conversely, it has been postulated that the suppression of the putative tumor suppressor gene *IGFBP3 *could lead to elevated levels of insulin-like growth factors, thus promoting tumor growth. Because mutational inactivation has been precluded as being causative for *IGFBP3 *suppression [16], epigenetic inactivation by promoter methylation has recently been considered as an alternative mechanism [17,18]. It is a well-described phenomenon that the suppression of tumor suppressor genes could be facilitated by abnormal methylation of DNA at certain CpG islands that often lie in the promoter regions of these genes [19].

Because the activation of IGF signaling is characteristic for HB and IGFBP3 suppression contributes to the sustainment of IGF signaling, we wanted to determine the role of the *IGFBP3 *gene in the biology of pediatric liver cancers. We demonstrate that the downregulation of *IGFBP3 *expression is a common feature in HB, which is associated with CpG island promoter methylation in advanced, high-risk HB cases. In addition, we reveal that *IGFBP3 *is epigenetically silenced in HB cell lines and that the reintroduction of *IGFBP3 *leads to the inhibition of tumor cell migration and invasion. These findings indicate that the suppression of IGFBP3 displays an alternative mechanism for enhancing IGF signaling in the late stages of HB development.

## Results

### Downregulation of *IGFBP3 *is a common event in pediatric liver tumors

**Figure 1:**
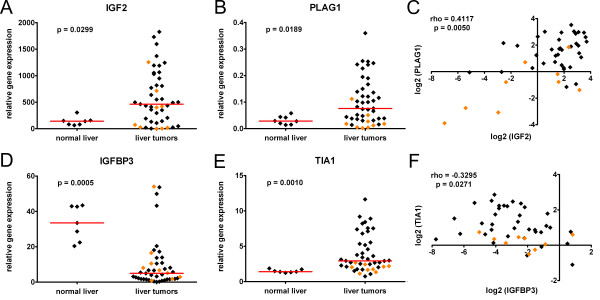
**Expression analyses of pediatric liver cancers by real-time PCR**. The relative *IGF2 ***(A) ***PLAG1 ***(B)**, *IGFBP3 ***(D)**, and *TIA1 ***(E) **mRNA expression levels of the 45 pediatric liver tumor cases and 7 normal liver samples were determined. The dots represent relative candidate gene expression in relation to the house-keeping gene *TBP*. Data from the HB and pediatric HCC cases are depicted as black and orange diamonds, respectively. Median expression values are given as red horizontal lines. **Correlation analyses of pediatric liver cancers**. The mRNA expression levels of *IGF2 ***(C) **and *IGFBP3 ***(F) **were correlated with *PLAG1 *and *TIA1*, respectively. Dots represent log_2 _values of the relative expression of candidate genes blotted versus each other in the 45 pediatric liver tumor samples (rho = Spearman's rank correlation coefficient).

To define the IGF signaling status in our pediatric liver tumor collection, we initially investigated the endogenous expression of the ligand IGF2 and its positive regulator PLAG1. Real-time PCR analysis revealed that the mRNA level of *IGF2 *was markedly increased (> 3-fold induction compared to normal livers) in 23/36 (64%) of HB and 3/9 (33%) of hepatocellular carcinoma (HCC) cases (Figure 1A). Furthermore, we detected a strong upregulation of *PLAG1 *in 20/36 (56%) of HB and 1/9 (11%) of HCC tumors (Figure 1B). Interestingly, a high *IGF2 *expression correlated well with *PLAG1 *upregulation, predominantly in HB cases (Figure 1C).

Because IGFBP3 has been described to act as a negative regulator of the IGF axis by competitively binding IGFs [11], we were interested in whether the downregulation of this gene could also contribute to the activation of IGF signaling in HB. By using real-time PCR, we demonstrate that *IGFBP3 *mRNA levels are heavily decreased (> 3-fold reduction compared to normal livers) in 26/36 (72%) of HB cases (Figure 1D). As previously described for HCC in adults [17], we also detected a reduced *IGFBP3 *expression in 6/9 (67%) of pediatric HCC cases (Figure 1D) compared to normal childhood liver tissues. *IGFBP3 *has recently been described to be transcriptionally downregulated by binding T-cell-restricted intracellular antigen-1 (TIA1), which is also overexpressed in human HCC [20]. Correspondingly, *TIA1 *is also upregulated in the majority of HB cases (Figure 1E) and is inversely correlated with the expression of *IGFBP3 *(Figure 1F), although at a low level (rho = -0.3295) Altogether, these data suggest that the downregulation of *IGFBP3 *might significantly contribute to the activation of the IGF signaling cascade by sustaining the IGF2-induced stimulation in HB.

### Promoter methylation causes *IGFBP3 *silencing in human HB cell lines

**Figure 2:**
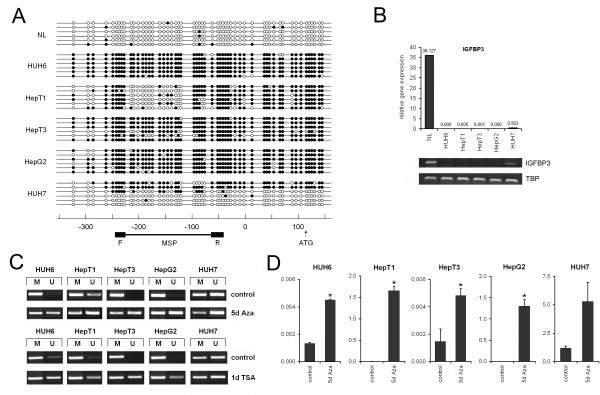
**(A) Methylation status of the *IGFBP3 *promoter in HB cell lines**. Bisulfite sequencing of the 5' region of *IGFBP3 *was performed in the indicated liver tumor cell lines and normal liver (NL) tissue. The open and filled circles represent unmethylated and methylated CpG sites, respectively. The location of each CpG site relative to the transcription start site (in base pairs) and the region used for MSP analysis, including the forward (F) and reverse (R) primer, is shown below. **(B) mRNA level of *IGFBP3 *in HB cell lines**. Expression of *IGFBP3 *was measured in normal liver tissue (NL) and in the liver tumor cell lines HepG2, HepT1, HepT3, HUH6, and HUH7 by real-time and RT-PCR. The values represent the relative gene expression in relation to the house-keeping gene *TBP*. **(C) Demethylation of the *IGFBP3 *promoter region after 5-Aza-dC or trichostatin A treatment**. Cells were incubated for 5 days with 5-Aza-dC and 24 hours with trichostatin A. Methylation status (U, unmethylated; M, methylated) of the *IGFBP3 *promoter region was determined using MSP. Representative images of the MSP experiments are given. **(D) Restoration of *IGFBP3 *expression**. The reactivation of *IGFBP3 *expression after 5 days of 5-Aza-dC treatment in HB cell lines, as revealed by real-time PCR. Statistically significant difference versus untreated cells: *P < 0.05 (unpaired Student's *t*-test).

Promoter methylation has been described as a molecular mechanism to suppress the gene expression of negative regulators of tumor growth in a variety of cancers [19]. Because TIA1 upregulation does not completely explain the suppression of *IGFBP3 *in pediatric liver tumors (Figure 1F), we examined a CpG island located in the *IGFBP3 *promoter region for differential methylation in established HB cell lines, namely HUH6, HepT3, HepT1, and HepG2, and the non-hepatitis B virus-associated HCC cell line HUH7, as well as normal liver by means of bisulfite sequencing. We found that the entire *IGFBP3 *promoter region was heavily methylated in all four HB cell lines and heterogeneously methylated in HUH7, whereas the normal liver DNA was rarely methylated in this region (Figure 2A). Interestingly, promoter methylation was well correlated with very low *IGFBP3 *expression levels in HB cell lines and a detectable expression in HUH7 when compared to a normal liver, as revealed by real-time and RT-PCR (Figure 2B). Because promoter methylation has a strong impact on the transcriptional activity, we next wanted to determine whether treatment with the demethylating agent 5-Aza-dC could revert the methylation status of the *IGFBP3 *promoter region and re-establish *IGFBP3 *expression in these cell lines. After the 5-day 5-Aza-dC treatment and subsequent MSP analysis, we detected an increasing amount of demethylation in the *IGFBP3 *promoter, thereby qualifying MSP as an appropriate means to analyze DNA methylation (Figure 2C). Bisulfite sequencing of single clones of 5-Aza-dC-treated HepG2 and HUH6 cells revealed a decreased methylation rate of 12.2% and 12.0%, respectively (data not shown). Interestingly, 5-Aza-dC treatment significantly re-established *IGFBP3 *expression in all cell lines (Figure 2D), which was most prominent in the HepT1 and HepG2 cells. These data suggest that promoter hypermethylation is causatively associated with transcriptional silencing of the *IGFBP3 *gene in pediatric liver tumors.

The histone-deacetylase inhibitor trichostatin A has formerly been described to display strong effects on the transcriptional regulation of IGFBP3 [21]. Treatment of all five liver cancer cell lines with trichostatin A resulted in the strong demethylation (Figure 2C) and reexpression (data not shown) of *IGFBP3*, comparable to the effect communicated by 5-Aza-dC but in a much shorter period (24 h). Thus, it might be expected that both promoter methylation and histone-deacetylation may play important roles in the control of the IGFBP3 tumor suppressor in the liver.

### *IGFBP3 *promoter methylation predominantly occurs in metastatic high-risk liver tumors with large vessel invasion

**Figure 3:**
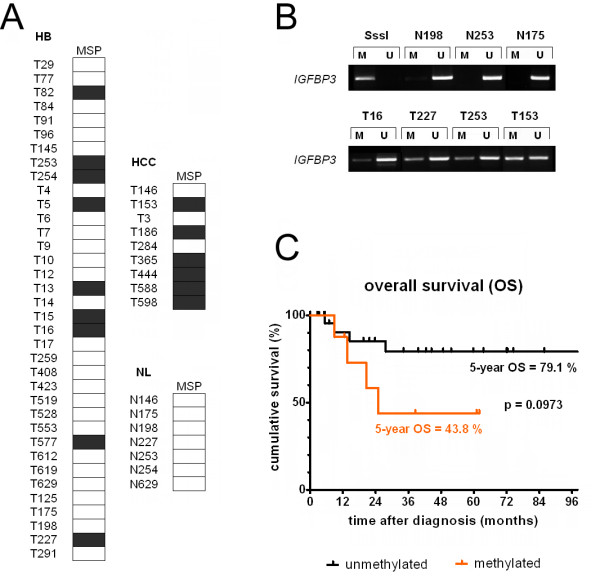
**(A) Methylation status of the *IGFBP3 *promoter in pediatric liver cancers**. Summary of the analyses of the *IGFBP3 *methylation status in the 36 hepatoblastomas (HB), 9 pediatric hepatocellular carcinomas (HCC), and 7 normal pediatric liver samples (NL), as determined by MSP. The black boxes represent the detection of methylated alleles, and the white boxes depict unmethylated alleles. **(B) Methylation-specific PCR (MSP) of pediatric liver cancers**. The promoter region of the *IGFBP3 *gene was analyzed for methylated CpG sites by MSP using bisulfite-treated genomic DNA from pediatric liver tumors (T) and normal liver tissue (N). Representative images of the MSP experiments are shown, revealing PCR products for unmethylated (U) and methylated alleles (M). *In vitro *methylated DNA (SssI) was used as a positive control for methylated alleles. **(C) Overall survival (OS) stratified by methylation status**. OS was calculated as the time from diagnosis to death from disease and is plotted for 8 HB patients with (orange line) and 25 without (black line) *IGFBP3 *promoter methylation over a period of 100 months. Statistical significance was calculated using Mantel-Cox test.

To assess whether *IGFBP3 *promoter methylation is clinically relevant, we performed a methylation analysis of our pediatric liver tumor collection using MSP. *IGFBP3 *methylation was detected in 9/36 (25%) of HB and 6/9 (66%) of pediatric HCC cases, whereas normal liver tissues had no bands for the methylated state (Figure 3A, B). However, there was no clear correlation between *IGFBP3 *promoter methylation and reduced *IGFBP3 *expression levels (data not shown). By analyzing clinicopathological features, such as gender, age at diagnosis, tumor differentiation, metastatic disease, outcome, multifocality, and vascular invasion (Table 1), we observed that *IGFBP3 *promoter methylation was significantly associated with metastases and invasion into large hepatic veins, two high-risk parameters for HB patients. Moreover, the overall survival of patients with *IGFBP3 *methylation was strongly reduced (Figure 3C). These data suggest that aberrant CpG island methylation of the *IGFBP3 *promoter region is a late event in the genesis of pediatric liver tumors and might predict the evolution of HB to a highly aggressive, metastatic, and vascular-invasive phenotype with worse outcomes.

**Table 1:** Associations of the clinical characteristics and *IGFBP3 *methylation status in 36 pediatric patients with hepatoblastoma

		IGFBP3			
				
parameters	group	no. of tumors	methylated	unmethylated	*P*
gender^a^	m	18	3	15	0.2482
	f	18	6	12	
age at diagnosis^b^	< 2	21	4	17	0.3570
	> 2	12	4	8	
histological subtype^c^	f	20	3	17	0.1213
	e	16	6	10	
metastases	yes	15	7	8	**0.0047**
	no	19	1	18	
outcome^d^	NED	25	4	21	0.0508
	DOD	8	4	4	
multifocal growth	yes	7	3	4	0.2662
	no	19	4	15	
invasion of large hepatic veins	yes	8	6	2	**0.0001**
	no	25	2	23	

^a^m, male; f, female

^b^The age of patients at diagnosis is given in months

^c^Epithelial component with predominantly fetal, f, or embryonal, e, differentiation

^d^NED, no evidence of disease; DOD, dead of disease

### Restoring IGFPB3 has long-term effects on cell growth and apoptosis in HB

**Figure 4:**
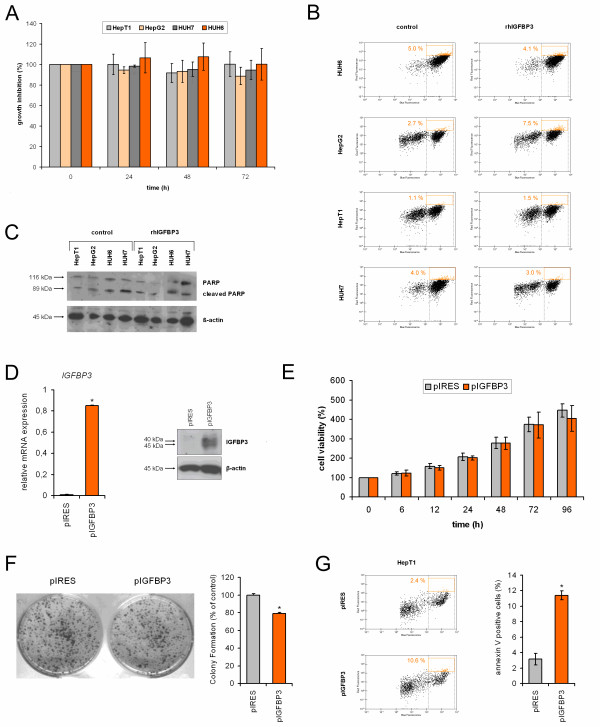
**(A) Growth properties of *IGFBP3 *treated HB cells**. The viability of tumor cells in the presence (IGFBP3) or absence (control) of 1 μg/ml recombinant human IGFBP3 was assessed at the time points indicated using MTT assays and optical density (OD) measurements. The values given represent the mean ratio of treated versus untreated cells from triplicate measurements ± SEM. **(B) Annexin V staining**. *IGFBP3*-treated cells were analyzed for phosphatidylserine membrane asymmetry using Cy5-conjugated annexin V and calcein staining. Representative plots of the flow-cytometric measurements depicting the percentages of apoptotic cells (annexin V and calcein positive) in the control (left panel) and recombinant human IGFBP3 treated cells (right panel) are shown. **(C) Proteolytic cleavage of PARP**. Activation of PARP by proteolytic cleavage was measured in tumor cells treated with or without recombinant human IGFBP3 by Western blot analysis using antibodies for cleaved PARP and β-actin as a loading control. **(D) Stable *IGFBP3 *transfectants**. HepT1 cells were transfected with an empty vector control (pIRES) or expression vector containing full-length *IGFBP3 *cDNA (pIGFBP3), selected with puromycin for 2 weeks and cloned by picking resistant colonies. Exogenous *IGFBP3 *expression was measured in transfected HepT1 cells using real-time PCR in relation to the house-keeping gene *TBP *as a calibrator **(left) **or Western blot analysis using antibodies for IGFBP3 and β-actin as a loading control **(right)**. **(E) Growth properties of *IGFBP3 *transfected HepT1 cells**. The viability of stably transfected cells was assessed at the time points indicated using MTT assays and optical density (OD) measurements. The values given represent the mean of triplicate measurements ± SEM. **(F) Colony formation assay**. HepT1 cells were transfected with an empty vector control (pIRES) or *IGFBP3 *expression vector (pIGFBP3) and subsequently cultured in puromycin-containing media for 2 weeks. Colonies were stained with crystal violet, and representative assays were photographed **(left) **and counted **(right)**. Statistically significant difference versus control: *P < 0.05 (unpaired Student's *t*-test). **(G) Annexin V staining**. *IGFBP3 *transfected cells were analyzed for phosphatidylserine membrane asymmetry using Cy5-conjugated annexin V and calcein staining. Representative plots of flow-cytometric measurements depicting 2.4% and 10.6% of apoptotic cells (annexin V and calcein positive) in the control (upper plot) and *IGFBP3 *transfectants (lower plot) are shown **(left)**. The mean percentages of early apoptotic cells ± SEM of two independent annexin V experiments are given **(right)**. Statistically significant difference versus vehicle: *P < 0.05 (unpaired Student's *t*-test).

IGFBP3 is thought to mediate growth suppression and induce apoptosis by binding IGFs [11]. Thus, we determined whether the reintroduction of IGFBP3 into liver tumor cells could change the tumor's biological properties. Adding 1 μg/ml recombinant human IGFBP3 to tumor cell lines resulted in comparable growth rates over time (Figure 4A). In line with this, IGFBP3-substituted cells displayed no significant increase in apoptotic characteristics, such as elevated external appearance of phosphatidylserine (Figure 4B) or proteolytic cleavage of the PARP protein (Figure 4C). In order to see long-term effects, we used HepT1 cells stably transfected with an *IGFBP3 *expression plasmid that resulted in highly elevated *IGFBP3 *mRNA and protein levels (Figure 4D). Although stable transfectants displayed no reduction in growth within 96 h (Figure 4E), we found a significantly reduced clonogenic survival rate after 2 weeks, as evidenced by the lower number of colonies (Figure 4F). Furthermore, *IGFBP3*-transfected cells showed signs of apoptosis, such as cell shrinkage, membrane blebbing, and formation of apoptotic bodies, when compared to control-transfected cells (not shown) and an increase in the external appearance of phosphatidylserine (Figure 4G). Taken together, our results document that long-term reconstitution of IGFBP3 acts as a tumor suppressive factor in pediatric liver tumors.

### Recombinant IGFBP3 slows the migratory and invasive capacity of liver tumor cells

**Figure 5:**
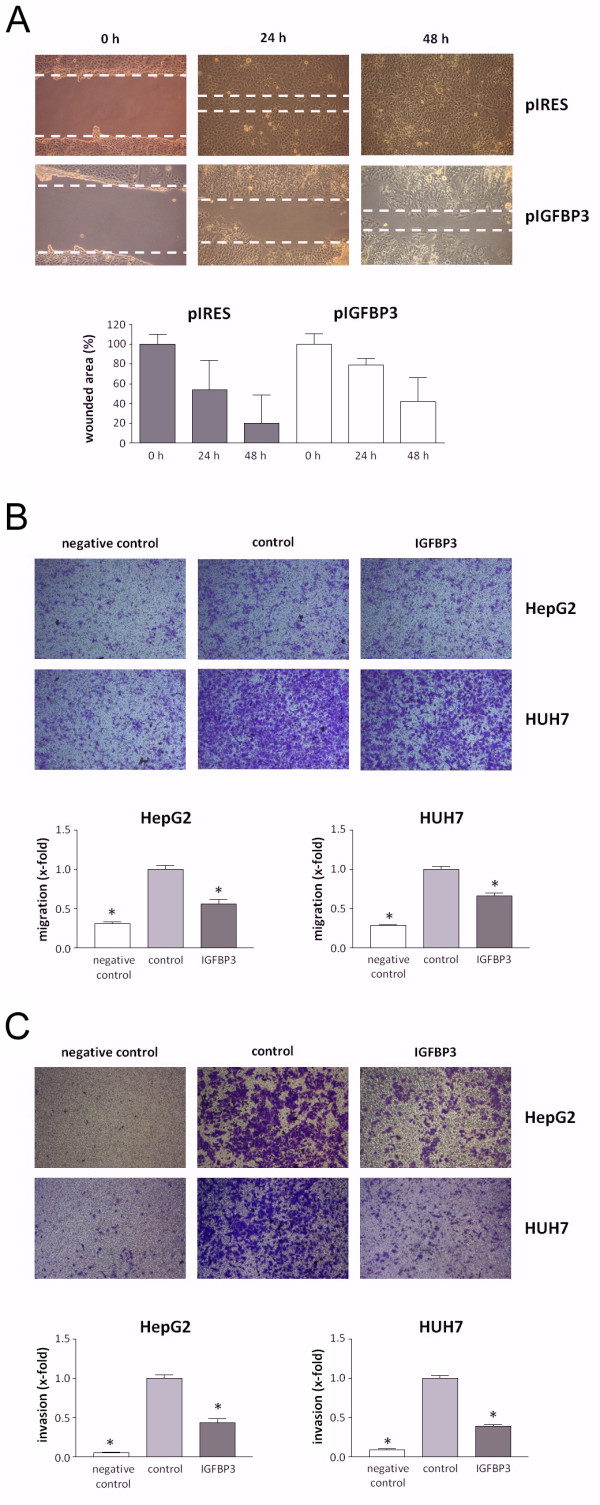
**(A) Scratch assay**. Representative images of HepT1 cells stably transfected with empty vector control (pIRES) or *IGFBP3 *migrating into a cell-free wound at 0, 24, and 48 h after wound infliction. Quantitative evaluation of wound closure shows the mean values of three independent experiments. **(B) Cell migration and (C) invasion assays**. Representative images of HepG2 and HUH7 cells migrating through collagen- **(B) **or Matrigel-coated **(C) **transwell plates (pore size, 8 μm) in the presence (IGFBP3) or absence (control) of 1 μg/ml recombinant human IGFBP3. 10% FCS and 50 ng/ml recombinant human HGF served as a chemoattractant in the lower compartment (IGFBP3 and control), whereas a negative control was run without a chemoattractant. The data are the mean ± SEM from three individual experiments. An asterisk indicates P < 0.05.

As IGFBP3 has been described to suppress migration and invasion in several cancers [22,23], we desired to determine whether the restoration of IGFBP3 function has any impact on the migratory and invasive capacity of liver tumor cells. Using wound healing assays, we demonstrated that HepT1 cells stably transfected with *IGFBP3 *had a markedly slower cell migration into a cell-free wound within 48 h than their control transfected counterparts (Figure 5A). By choosing liver tumor cell lines with high migration rates, namely HepG2 and HUH7, migration assays using collagen-coated transwell inserts demonstrated a significantly decreased migration of tumor cells incubated with recombinant human IGFBP3 (Figure 5B). Moreover, tumor cells lost their invasiveness when recombinant human IGFBP3 was added to the culture medium, as evidenced by the transwell assays with Matrigel coated inserts (Figure 5C). Altogether, these data clearly indicate that restoring IGFBP3 function could dramatically diminish the migratory and invasive properties of liver tumor cells.

## Discussion

Binding of the IGF2 ligand and the subsequent activation of the IGF1 receptor is known to confer a survival advantage for a wide range of cell types [24]. Consequently, constitutive activation of the IGF axis is a common feature of tumor cells, especially those of early childhood cancers [6,25]. The prevailing mechanism for IGF pathway activation in HB has been allocated to the overexpression of *IGF2*, which is a result of genetic and epigenetic alterations at the *PLAG1 *and *IGF2/H19 *locus [7,8,10,26] and causes activation of the downstream serine/threonine kinase and survival factor AKT [27]. The present study adds an alternative activation mechanism, namely the augmentation of the IGF/IGF1R interaction through downregulation of the IGF2 competitor IGFBP3. We provide evidence that low *IGFBP3 *expression is a common phenomenon in HB that may contribute to the activation of the IGF axis at the physiological level by the loss of ligand sequestration. Furthermore, the loss of *IGFBP3 *expression could be attributed to the methylation of the *IGFBP3 *promoter in at least some primary HB cases, with a predominant occurrence of this epigenetic alteration in metastatic and vascular-invasive high-risk tumors. Our data support the hypothesis that *IGFBP3 *silencing may contribute to enhanced IGF2/IGF1R signaling and thus the survival and progression of transformed liver cells at a late stage of the disease, which may eventually have considerable clinical implications.

One interesting finding of the current study is that promoter hypermethylation is one possible mechanism for *IGFBP3 *silencing in HB. We unequivocally demonstrated that DNA is heavily methylated throughout the entire *IGFBP3 *promoter region of all four HB cell lines under investigation, which conveys a strong suppression of *IGFBP3 *transcription. These repressive modifications could be removed by the addition of the demethylating agent 5-Aza-dC to the cycling cells, thereby re-establishing *IGFBP3 *expression. Aberrant DNA methylation has been shown to play an important role in the silencing of *IGFBP3 *expression in several human cancers, including gastric, colorectal, breast [18], ovarian [28], and renal cancer [29], as well as HCC in adults [17]. However, because DNA methylation only explains the downregulation of *IGFBP3 *in a subset of primary HB cases, molecular mechanisms other than DNA methylation might also be responsible for the low *IGFBP3 *expression levels found in the majority of primary HB tumors. Degradation of IGFBP3 by cathepsin D, a specific protease of IGFBP3, has been envisaged as an alternative suppression mechanism of IGFBP3, at least at the protein level [17]. Upregulation of the regulatory protein TIA1 that binds to the AU-rich region of the 3'-UTR of *IGFBP3 *has recently been described to be associated with downregulation of *IGFBP3 *in primary HCC [20]. As we have detected an inverse correlation of *TIA1 *and *IGFBP3*, it could be assumed that this suppressive mechanism could act in pediatric liver tumors. In addition, histone deacetylation may also play an important role in the suppression of IGFBP3, as shown in this and other studies [21]. Nevertheless, technical restrictions, such as heterogeneity of tumor samples, which comprise the stromal components and the adjacent normal liver tissue in low proportions, might have contributed to an underestimation of HB cases with a methylated *IGFBP3 *promoter in our study. Noteworthy, a discrepancy between high methylation rates in tumor cell lines and relative low rates in primary tumors is a common phenomenon [30-32]. It has been suggested that a large proportion of CpG hypermethylation found in cancer cell lines reflects an intrinsic property of mammalian cells grown in culture rather than a dependency on the cell of origin. Furthermore, the accumulation of epigenetic changes during the prolonged culture of human embryonal stem cell lines and their derivatives has been described [33]. Alternatively, it might be speculated that subclones within primary cancers with aberrant CpG island methylation may be preferentially selected during cell passage and/or that cancers with high levels of aberrant CpG methylation could be more likely to become established as cell lines. Nevertheless, our functional data clearly show that IGFBP3 silencing is not just a cell culture artifact, but instead, it plays an important role in driving adverse growth characteristics of liver cancer cells originating from advanced stages of liver tumor development.

In addition to its mechanistic role in gene silencing, *IGFBP3 *promoter methylation might also have clinical implications as a biomarker. It has been reported that *IGFBP3 *is frequently methylated and significantly associated with a poor prognosis in early-stage non-small-cell lung [34,35], ovarian [28], and prostate cancer [36]. In contrast to these studies, in which hypermethylation of the *IGFBP3 *promoter is a common and early event during tumorigenesis, we found only 9/36 of HB tumor cases to be methylated, seven of which were high-risk metastatic tumors, indicating a late event in the development of HB. Moreover, as *IGFBP3 *promoter methylation was significantly associated with vascular invasion in HB and occurred more frequently in pediatric HCC, the detection of this epigenetic alteration might be used as an attractive biomarker for stratifying patients for risk-adapted therapy. Congruent with our assumption, high promoter hypermethylation frequencies of tumor suppressor genes, including *IGFBP3*, already serve as an indicator for a distinct subclass of advanced HCC in adults with a poor prognosis [37]. This relationship, in turn, suggests that demethylating drugs, which have already been under clinical evaluation [38], might be a novel therapeutic option to treat high-risk liver tumor patients. However, further studies in a large cohort of HB patients are warranted.

Our finding that IGFBP3 restoration results in reduced tumor cell migration and invasion, while leaving growth and apoptosis merely unaffected, also underscores the assumption that *IGFBP3 *acts at more advanced stages of liver tumor development in children. Furthermore, IGFBP3 has been shown to suppress migration and invasion in adult HCC [22] and melanoma [23]. Interestingly, low IGFBP3 levels have been found to correlate with higher portal invasion and worse prognosis in HCC [39]. Altogether, these data suggest that IGFBP3 downregulation likely has a major role in the vascular invasive and metastatic growth properties of pediatric liver tumors.

## Conclusions

In summary, our study clearly documents the following regarding *IGFBP3*: i) it is downregulated in a high proportion of pediatric liver tumors; ii) it is epigenetically silenced in a subset of HB, indicating that additional repressive mechanisms must exist for this gene; iii) promoter methylation is a late event and predominantly occurs in progressed metastatic and vessel-invasive HB, which may be of clinical significance for HB patients by proposing adapted therapies; and iv) it prevents the migration and invasiveness of HB. Thus, it is intriguing to speculate that restoring *IGFBP3 *expression and/or use of demethylating drugs could contribute to new therapeutic strategies for HB, especially with the existence of additional epigenetically silenced genes in this tumor type, such as *HHIP, RASSF1, SOCS1, APC *and *CASP8 *[40].

## Methods

### Subjects and tumor cell lines

A total of 45 liver tumor specimens were obtained from pediatric patients undergoing surgical resection in our clinic. Normal liver matching was available from seven patients (N146, N175, N198, N227, N253, N254, and N629). Written informed consent was obtained from each patient, and the study protocol was approved by the Committee of Ethics of the Ludwig-Maximilians-University of Munich. We used the HB cell lines HUH6 (Japanese Collection of Research Bioresources, JCRB, Osaka, Japan), HepT1 [41], HepT3 [7], and HepG2 [42], as well as the hepatocellular carcinoma (HCC) cell line HUH7 (JCRB). All cell lines were maintained as the suppliers recommended.

### Real-time reverse transcription-PCR (RT-PCR)

The total RNA was extracted from macroscopically dissected frozen tumor tissue (at least 80% tumor cells), frozen normal liver tissue and HB cell lines, depleted from residual DNA, and reverse transcribed as previously described [43]. PCR amplifications were carried out with 40 ng of cDNA, 500 nM forward and reverse primers and iTaq SYBR Green Supermix (Bio-Rad Laboratories, Hercules, CA, USA) on a Mastercycler Realplex^2 ^cycler (Eppendorf, Hamburg, Germany) with 40 cycles consisting of a 15 sec denaturation at 95°C, primer annealing for 15 sec at 55°C, and extension for 30 sec at 72°C. We used the following primer pairs (5'- > 3' orientation): *IGF2*, CCTCCGACCGTGCTTCC, GGTGGACTGCTTCCAGGTGT; *PLAG1*, ACAAGTGCATACAACAAGACTGCA, CAGGAGAATGAGTAGCCATGTGC; *IGFBP3*, GTCCAAGCGGGAGACAGAATAT, CCTGGGACTCAGCACATTGA; *TIA1*, TTAGCCAGATTGGACCTTGTAAAAA, CGATGCTCATGAAACTCCACA; *TBP*, GCCCGAAACGCCGAATAT, CCGTGGTTCGTGGCTCTCT. Amplification of the house-keeping gene *TATA-Box-binding-Protein *(*TBP*) was performed to standardize the amount of sample RNA according to a previous study [44]. PCR efficiencies for all assays were determined, with slopes ranging from -3.34 to -3.69. The relative quantization of gene expression was performed using the ΔΔct method as previously described [45].

### Methylation analyses

Genomic DNA from frozen tumor and normal liver samples (see above) and cell lines was extracted with phenol and chloroform, precipitated with ethanol and dissolved in TE buffer following standard procedures. Genomic DNA (10 μg) from a healthy person was methylated *in vitro *using 40 U CpG methyltransferase (SssI), S-adenosylmethionine, and NEBuffer2 (New England Biolabs, Frankfurt, Germany) at 37°C for 4 h, precipitated with ethanol, dissolved in TE buffer, and used as a positive control for methylated alleles. Genomic DNA (2 μg) was bisulfite-treated using the EpiTect^® ^Bisulfite Kit (Qiagen, Hilden, Germany) and amplified using primers not specific for methylation status (IGFBP3-BS-F, GGTGTTGAGTTGGTTAGGAGT; IGFBP3-BS-R, AAACAACACCAACAAAATCAA). We cloned the PCR products into the pCR2.1 TOPO vector (Invitrogen, Karlsruhe, Germany) and sequenced six independent clones per sample (MWG Biotech, Ebersberg, Germany).

In addition, the methylation status of the promoter region of *IGFBP3 *(from -314 to -147 bp upstream of transcriptional start site) gene was analyzed by methylation-specific-PCR (MSP) using the following primer sets 5'- > 3' orientation): methylated (IGFBP3-M-F, TGATTCGGGTTTCGGGCGTGC; IGFBP3-M-R, GCCGACCGCTATATAAAAACCG) and unmethylated (IGFBP3-U-F, GGTGATTTGGGTTTTGGGTGTGTGTAT; IGFBP3-U-R, AAACACACCAACCACTATATAAAAACCAAA). MSP primer design and PCR conditions were performed according to [43]. For DNA demethylation experiments, we used 0.5 μM 5-aza-2'-deoxycytidine (5-Aza-dC; Sigma-Aldrich, Seelze, Germany) for HUH6 and HepT3 cells and 1.25 μM for HepT1, HepG2 and HUH7 cells; 5-the Aza-dC was applied for 5 days and changed daily. Alternatively, Trichostatin A (Sigma-Aldrich) was applied for 24 h in a concentration of 0.1 μM (HUH6 and HepT3) and 0.25 μM (HepT1, HepG2 and HUH7).

### Stable transfection

HepT1 cells (5 × 10^5 ^cells/6-well plate) were transfected with 1 μg DNA of the pIRES-IGFBP3 expression vector containing full-length *IGFBP3 *cDNA [46] or the empty vector control using the FuGene 6 transfection reagent (Roche Diagnostics, Mannheim, Germany). After 24 h of transfection, the cells were changed to media containing 1 μg/ml puromycin (Sigma-Aldrich). After 2 weeks of selection, puromycin-resistant colonies were selected and cultured as stable transfected HepT1 clones. Western blot analysis was performed using rabbit polyclonal anti-IGFBP3 (Santa Cruz Biotechnology, Santa Cruz, CA, USA) and rabbit anti-human β-actin (Cell Signaling, Technology, Danvers, MA, USA) antibodies, as previously described [43].

### Cell viability assay

For the proliferation assay, 5 × 10^3 ^cells were seeded into 96-well plates, and the viability was assessed at the time points indicated using the Cell Proliferation Kit I (Roche Diagnostics) according to the manufacue's protocol. The optical density was measured at a wavelength of 595 nm after the addition of 3-(4,5-dimethylthiazol-2-yl)-2,5-diphenyltetrazolium bromide (MTT) labeling reagent on the GENios microplate reader (Tecan, Männedorf, Switzerland).

### Colony formation assay

HepT1 cells (5 × 10^5 ^cells/well) were transfected in a 6-well plate format with 1 μg of the pIRES-IGFBP3 expression vector or control vector using the FuGene 6 transfection reagent (Roche Diagnostics). They were subsequently cultured in selection media containing 1 μg/ml puromycin (Sigma-Aldrich) for 2 weeks. Colonies were fixed with 100% methanol, stained with 0.1% crystal violet and counted.

### Apoptosis analyses

For annexin V-based apoptosis analysis, cells were trypsinized, washed with PBS, and suspended in 500 μl of calcium-containing binding buffer. Cy5-conjugated annexin V (1:100; BioVision, Mountain View, CA, USA) and 5 μM calcein (Invitrogen) were added to the cell suspension. Early apoptotic cells (annexin V and calcein positive) were detected using cell fluorescence assays with an Agilent 2100 Bioanalyzer. The cleavage of poly(ADP-ribose) polymerase was detected as previously described [47] using antibodies for human poly(ADP-ribose) polymerase and human β-actin (both from Cell Signaling Technology, Danvers, MA, USA).

### Cell migration assay

HB cells were seeded into 24-well plates and grown to confluency. A wound of approximately 1 mm was inflicted to cell monolayers with a pipette-tip. The wells were washed twice with PBS to remove detached cells and incubated at 37°C with medium in the presence or absence of 1 μg/ml recombinant human IGFBP3 (R&D systems, Wiesbaden, Germany) for 72 h. Images were taken at 0, 24, and 48 h after scratching, and the wound widths were measured and quantified.

### Transwell assays

Transwell permeable supports (8 μm pore polycarbonate inserts; Corning Incorporated, Corning, NY, USA) were coated either with collagen or 10% Matrigel^® ^(BD Biosciences, Heidelberg, Germany) in DMEM and subsequently added to 24-wells containing DMEM (negative control) or DMEM/10% FCS/50 ng/ml recombinant human HGF (NatuTec, Frankfurt, Germany) as a chemoattractant. Cells (1 × 10^5^) were seeded in DMEM in the inside compartments and allowed to migrate for 16 h (HUH7) or 72 h (HepG2) in the presence or absence of 1 μg/ml recombinant human IGFBP3 (R&D systems). Afterwards, the inserts were stained with crystal violet solution. Cells from the upper side of the insert were removed by using cotton swabs. Cells attached to the bottom side of the insert were photographed using a Zeiss Axiovert 25 microscope and a Canon 450D camera. For each sample, eight pictures were taken, and the number of cells was calculated by using ImageJ (National Institute of Health, Bethesda, MD, USA) and the Particle Counter plugin. Data for three independent experiments were analyzed using GraphPad Prism Version 3.0 (GraphPad Software, La Jolla, CA, USA).

### Statistical analysis

The data are presented either as dot plots or bar graphs, indicating the mean ± SEM. The statistical analyses and Kaplan Meier calculations were performed with GraphPad Prism Version 3.0 using Student's unpaired *t*-test, Mantel-Cox test, Mann-Whitney-*U *test, Spearman's rank correlation, one-way ANOVA and Dunnett's test. *P *< 0.05 was considered to be significant.

## Abbreviations

HCC: Hepatocellular carcinoma; HB: Hepatoblastoma; IGF2: Insulin-like growth factor 2; IGFBP3: Insulin-like growth factor binding protein 3; MSP: Methylation-specific-PCR; PLAG1: Pleomorphic adenoma gene 1; RT-PCR: reverse transcription-PCR; TBP: TATA-Box-binding-Protein; TIA1: T-cell-restricted intracellular antigen-1; 5-Aza-dC: 5-aza-2'-deoxycytidine.

## Competing interests

The authors declare that they have no competing interests.

## Authors' contributions

IR carried out the processing of clinical samples, expression measurements, migration assays, 5-Aza-dC treatments, and the methylation-specific PCR, and she participated in the analysis and interpretation of the data. ME performed the Western blot and apoptosis assays. SJ conducted the bisulfite-sequencing study and interpreted the methylation data. JL performed the invasion and migration assays. BH collected the clinical samples and carried out the statistical analyses of the clinical data. JMH performed the pathological diagnosis and staging of tumor specimens. AV participated in the design of the study and helped in the analysis and interpretation of the data. DvS participated in the design of the study and helped to draft the manuscript. RK designed the study, participated in the analysis and interpretation of data, and drafted the manuscript. All of the authors read and approved the final version of this manuscript.
